# Modulation of the monomer-dimer equilibrium and catalytic activity of SARS-CoV-2 main protease by a transition-state analog inhibitor

**DOI:** 10.1038/s42003-022-03084-7

**Published:** 2022-03-01

**Authors:** Nashaat T. Nashed, Annie Aniana, Rodolfo Ghirlando, Sai Chaitanya Chiliveri, John M. Louis

**Affiliations:** 1 Laboratory of Chemical Physics, National Institute of Diabetes and Digestive and Kidney Diseases, National Institutes of Health, Bethesda, MD USA; 2 Laboratory of Molecular Biology, National Institute of Diabetes and Digestive and Kidney Diseases, National Institutes of Health, Bethesda, MD USA

**Keywords:** Enzyme mechanisms, Mechanism of action, Proteases, Biophysical chemistry

## Abstract

The role of dimer formation for the onset of catalytic activity of SARS-CoV-2 main protease (MPro^WT^) was assessed using a predominantly monomeric mutant (MPro^M^). Rates of MPro^WT^ and MPro^M^ catalyzed hydrolyses display substrate saturation kinetics and second-order dependency on the protein concentration. The addition of the prodrug GC376, an inhibitor of MPro^WT^, to MPro^M^ leads to an increase in the dimer population and catalytic activity with increasing inhibitor concentration. The activity reaches a maximum corresponding to a dimer population in which one active site is occupied by the inhibitor and the other is available for catalytic activity. This phase is followed by a decrease in catalytic activity due to the inhibitor competing with the substrate. Detailed kinetics and equilibrium analyses are presented and a modified Michaelis-Menten equation accounts for the results. These observations provide conclusive evidence that dimer formation is coupled to catalytic activity represented by two equivalent active sites.

## Abstract (web summary)

The binding of a drug targeting the active site of a predominantly monomeric SARS-CoV-2 main protease (MPro^M^) favors an equilibrium shift to MPro^M^ dimer formation with two equivalent active sites. These results suggest targeting the monomeric active site and/or the dimer interface to interfere with the conformational rearrangements to active dimer formation as an alternative drug design strategy against MPro.

## Introduction

In Severe Acute Respiratory Syndrome CoronaVirus 2 (SARS-CoV-2), which causes the COronaVIrus Disease 2019 (COVID-19)^1,2^, and its closely related SARS-CoV, the function of the main protease (MPro) is indispensable for its replication and propagation^1,3,4^. In its genome, a single copy of MPro is encoded within the polyproteins (pp) 1a and 1ab^4,5^. The active MPro functions as a homodimer to mediate its own release at its termini and processing of the polyproteins at various sites to generate the non-structural proteins nsp4 through nsp16 required for the assembly of the viral replication/transcription complex^3,6,7^. Thus, in addition to effective vaccines targeting the spike protein^8^, MPro is a potential target for the development of antiviral agents for the treatment of SARS-CoV-2 infection^3,9,10^. An active site inhibitor (PF-7321332) of MPro is currently in clinical trials for the treatment of COVID-19^11^.

MPro is composed of 306 amino acids comprising three domains. Domains I (residues 8–101) and II (residues 102–184) together exhibit a chymotrypsin-like fold, and domain III (residues 201–306) comprises a cluster of five alpha-helices connected to domain II by a long loop (residues 185–200)^3,7^. Each subunit of MPro harbors an active site consisting of the catalytic dyad H41-C145. Cleavage at the N-terminus of MPro has been proposed to modulate the quaternary structure for catalytic activity through extensive inter- and intra-subunit contacts formed by the free N-terminal strand with domains II and III^3,7,12–15^. The released fully active wild-type mature MPro, henceforth referred to as MPro^WT^, exhibits a dissociation constant in the low micromolar range of 0.1 to 15^3,16–20^ capable of cleaving polyprotein and synthetic peptide substrates. Deletion of the N-terminal residues (termed the N-finger) and domain III lead to a shift in the monomer-dimer equilibrium towards the monomer form accompanied by a drastic decrease in catalytic activity^17,21–25^. Various mutational analysis of SARS-CoV MPro and structural requirements for its regulation are summarized in references^20,26^. Despite the monomer form adopting a native-like tertiary fold, as shown for various mutations or deletions in the sequence, monomeric variants of MPro are reported to exhibit very low or no catalytic activity^7,17,21,26–33^. This has been attributed to a collapsed active site which impairs the binding of Q-P1 of the substrate in the S1 subsite leading to loss of catalytic function^28,29,32^. Specifically, in the monomeric structure the loop comprising residues S139 to L141 was shown to transform into a 3_10_-helix such that the rearranged N142 interacting with E166 blocks entry to the S1 subsite. This is consistent with the observation that mutation E166A also impairs substrate binding^17,20^. Single mutations of R4, M6, G11, S139, E290, and R298 lead to increased dimer dissociation^20,27–29,31,32^. Specifically, inter-subunit contacts mediated by a salt bridge between residues R4 and E290^27^ and an aromatic-hydrophobic interaction between Y126 and M6, as well as an intra-subunit hydrogen bond between the side chain NH2 of R298 with backbone oxygen of M6, are shown to be essential for maintaining dimerization^20,28^. Thus, to closely examine the role of dimerization for the onset of catalytic activity, we took advantage of critical contacts mediated by residues E290 and R298 in domain III, mutations of which substituted to Ala lead to a significant increase in the dimer dissociation constant (K_d_ = K_1_). The resulting predominantly monomeric construct allowed for a detailed examination of the relationship between dimer formation and the kinetics and inhibition of MPro-catalyzed hydrolysis using the transition-state analog inhibitor GC376 that modulates the relative composition of the monomer and dimer form of the enzyme. These studies provide conclusive evidence for the role of dimer formation and its associated thermodynamic stability being pivotal for the appearance of mature-like catalytic activity, with two equivalent active sites.

## Results

### Preparation and characterization of MPro^M^

**Fig. 1:**
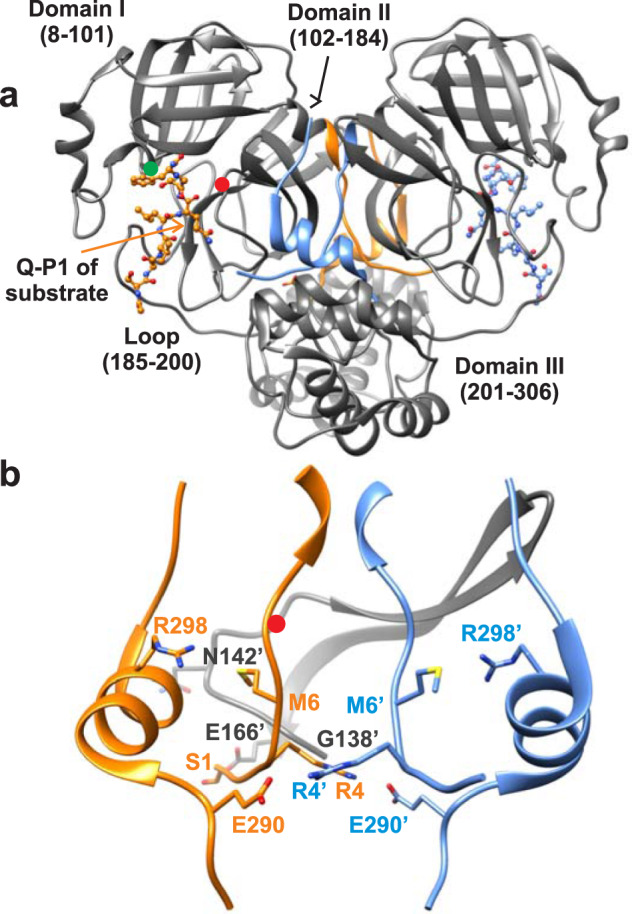
Molecular representation of the SARS-CoV-2 main protease dimer and critical interactions which influence the monomer-dimer (M-D) equilibrium. **a** The two subunits of the mature dimer (PDB ID: 7N89^54^) with N- (1–11) and C-terminal (288–306) residues highlighted with subunit A in orange and subunit B in blue. Substrate SAVLQSGF bound to the active site of each subunit is shown with Q-P1 which forms the S1 subsite indicated by the orange arrow. The interface formed by the free N-finger residues 1–7, shown in the middle of the dimer, is critical for dimer stability. The dimer interface is formed by an extensive network of hydrogen bonds and hydrophobic interactions involving N-terminal residues 1′-16′, β-strand residues 118′-125′ and loop residues 137′-142′ as illustrated in reference^15^. Red and green circles denote position of active site C145 and H41 residues, respectively. **b** Enlargement of the region showing residue positions with the same coloring as in (A) critical for dimer interface stability. Mutations E290A and R298A increase the K_d_ by ~5000-fold based on our estimate shown in Table 1, and published reports^3,17,18^. The region (G138 to E166) encompassing the oxyanion loop (S139 to L141) for subunit B are shown in gray. S1 of subunit A interacts with E166 of subunit B^17^. Residues from subunit B are denoted with prime (‘).

A mini precursor of MPro (termed ^+25^MPro^M^-6His) containing 25 amino acids of flanking nsp4 sequence, substitution mutations E290A and R298A in domain III and a C-terminal 6His-Tag was constructed (Fig. 1 and Supplementary Fig. 1). Consistent with an earlier observation of an analogous model precursor construct of MPro^WT^ or with the single R298E mutation in MPro^7^, expression of ^+25^MPro^M^-6His results in its maturation at the nsp4/nsp5 junction and accumulation of mature MPro^M^-6His. The 6His-Tag which permits facile purification was subsequently removed using human rhinovirus (HRV-3C) protease as described^34^ yielding MPro^M^ (Supplementary Fig. 1).

**Fig. 2:**
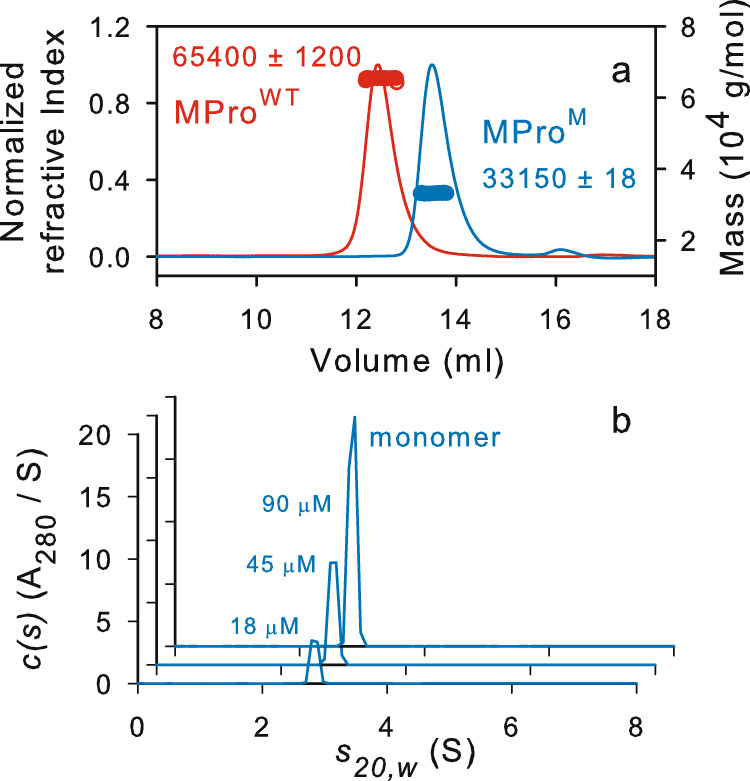
Molecular mass estimation of MPro^M^. **a** Mass estimation by SEC-MALS by injecting 125 µL of MPro^M^ and MPro^WT^ at ~58 µM and 30 µM, respectively. **b** SV-AUC absorbance *c(s)* distributions at concentrations ranging from 18 to 90 µM of MPro^M^. SEC-MALS and SV-AUC were carried out in buffer A at 25 °C.

Processed (mature) MPro^M^ was purified using established methods (Supplementary Fig. 1) from cells induced for expression for 2–3 h. MPro^M^ showed an estimated mass of 33653 Da by ESI-MS (calculated = 33654 Da) and a single peak corresponding to a monomer mass of 33.2 kDa by size exclusion chromatography coupled with multi-angle light scattering (SEC-MALS, Fig. 2a). Sedimentation velocity analytical ultracentrifugation (SV-AUC), which precludes dilution during the experiment, at concentrations ranging from 18–90 µM clearly shows that MPro^M^ is mainly monomeric with no detectable dimer form. As shown in Fig. 2b, a single species of 2.84 S with an estimated mass of 33 kDa was observed up to 90 µM. This result is consistent with such mutations introduced in SARS-CoV main protease influencing the M-D equilibrium (Fig. 1b)^7,17,26–28^. In contrast, mature MPro^WT^ elutes as a dimer exhibiting a mass of 65.4 kDa by SEC-MALS as expected (Fig. 2a).

### Kinetics of MPro^WT^ and MPro^M^-catalyzed hydrolyses

**Fig. 3:**
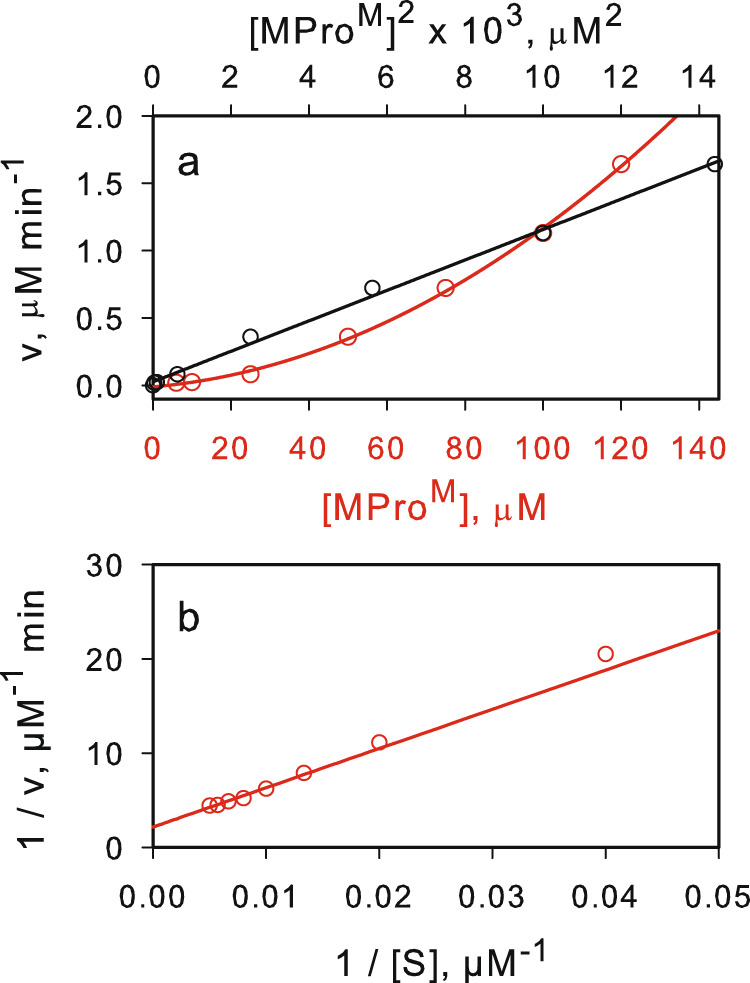
Evaluation of the catalytic efficiency of MPro^M^. **a** Non-linear relationship between the rate of catalyzed hydrolysis *vs* the protein concentration (red line), and the linear relationship between the rate of catalyzed hydrolysis *vs* the square of the protein concentration (black line). **b** Lineweaver-Burk plot for the catalyzed hydrolysis at a concentration of 40 µM MPro^M^.

MPro^WT^ and MPro^M^ catalyze the hydrolyses of a known fluorescence resonance energy transfer (FRET) peptide substrate^3,35–37^ corresponding to the nsp4/nsp5 cleavage site sequence in pp1a polyprotein^3^. The rates of hydrolyses at a final substrate concentration of 200 μM display linear relationships with the square of the concentration of MPro^WT^ and MPro^M^ with intercepts at the origin [multiple correlation coefficient (R) = 0.9972, Supplementary Fig. 2a and R = 0.9984, Fig. 3a, respectively]. Second-order dependency on protein concentration provides clear evidence that the dimeric form of the enzyme is required for catalytic activity. These observations are in accordance with an earlier report of the monomeric R290A and R290L mutants of SARS-CoV main protease^17^, which shares 95% sequence identity to MPro^WT 3^. The rates of MPro^WT^- and MPro^M^-catalyzed hydrolyses display good substrate saturation kinetics at constant protein concentrations (Fig. 3b and Supplementary Fig. 2b) and the resulting v_max_ and K_s_ are listed in Table 1 including the total initial monomer concentration (M_o_).

**Table 1:** Kinetic, thermodynamic and inhibition parameters of MPro^WT^ and MPro^M^-catalyzed hydrolysis of peptide substrate^a^.

Protein	M_o_ µM	K_1_ µM	V_max_ µM min^-1^	k_cat_ min^-1^	K_m_ = K_s_ µM	(k_cat_/K_m_)_obs_ µM^-1^ min^-1^	k_3_/K_s_ µM^-1 ^min^-1^	-ΔG kcal/mol	K_i_ µM
MPro^WT^	0.2	1.3 ± 0.2	2.1 ± 0.1	54 ± 3	91 ± 2	0.6 ± 0.05	0.6 ± 0.05	0.306	0.15^b^
MPro^M^	40	6600^b^	0.46 ± 0.04	0.97 ± 0.1	193 ± 29	(5 ± 0.6)10^-3^	5 × 10^-3^	3.2	
MPro^M^ + GC376	10					(1.3 ± 0.05)10^-3^	3.4 × 10^-3c^	3.4	6.2^d^

^a^Protease assays were carried out in buffer B (25 mM Tris-HCl, pH 7, 50 mM NaCl and 1 mM TCEP) at 28 °C. ^b^Determined by ITC. ^c^The value of k_cat_/K_m_ is calculated from the observed k_cat_/K_m_ at GC376 concentration of 10 µM and [M_o_] of 10 µM using the expression: $({k}_{{cat}}/{K}_{m})={k}_{3}/2{K}_{s}(1+[I]/{K}_{i})$ (see Eq. 4). ^d^The binding constant is the average of binding constants obtained from inhibition study, ITC, and SV-AUC. -ΔG values were calculated from the k_3_/K_s_ values.

**Fig. 4:**
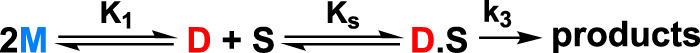
Mechanism of catalysis by MPro^WT^ and MPro^M^. M, D and S denote monomer, dimer and substrate, respectively.

Scheme in Fig. 4 is proposed to account for the observed results (see Supplementary Note 1).

It can be shown that:

1 $$v=\frac{{k}_{3}\left\{\left[{M}_{o}\right] \! - \! \left[M\right]\right\}\left[S\right]}{{K}_{s}+[S]}$$

where [*M*_o_] and [M] denote the total and free monomer concentrations, respectively. For MPro^WT^, K_1_ was determined by the kinetic method described previously (Supplementary Fig. 2c)^38,39^. The obtained K_1_ value is 1.32 ± 0.2 µM, which is consistent with the earlier reported estimate by SV-AUC under similar buffer conditions for MPro^WT 3,17^ and for SARS-CoV MPro^WT 3,17^ was used to calculate the free monomer concentration. A plot of v *vs* {[M_o_]-[M]} is linear (R = 0.9979, Supplementary Fig. 2d) with an intercept at the origin indicating that enzymatic activity is observed only from the dimeric form of MPro^WT^. The kinetic parameters and M-D equilibrium constant are listed in Table 1.

For MPro^M^, the value of K_1_ is much larger than 90 µM and thus could not be determined directly. Since [M_o_] ≈ [M]>>>[D], {[M_o_]-[M]} = 2[D], and [D] = [M]^2^/K_1_, substituting {[M_o_]-[M]} with the value of 2[D] and [M]^2^ with [M_o_]^2^ and rewriting Eq. 1 gives:

2 $$v=\frac{2{k}_{3}\left[S\right]\left\{\frac{{\left[{M}_{o}\right]}^{2}}{{K}_{1}}\right\}}{{K}_{s}+[S]}$$

As indicated above, a plot of v *vs* [M_o_]^2^ at substrate concentration of 200 µM is a straight line. From a plot of 1/v *vs* 1/[S] (Fig. 3b), the obtained K_s_ of 193 µM is about the substrate concentration used and thus, the slope of the line is:

3 $$\frac{{k}_{3}[S]}{{K}_{1}{K}_{s}}=(1.0\pm 0.03){10}^{-4}\,{\upmu {{{{{\rm{M}}}}}}}^{-1}{{{\min }}}^{-1}$$

### Modulation of the catalytic activity of MPro^M^ by feline coronavirus prodrug GC376

**Fig. 5:**
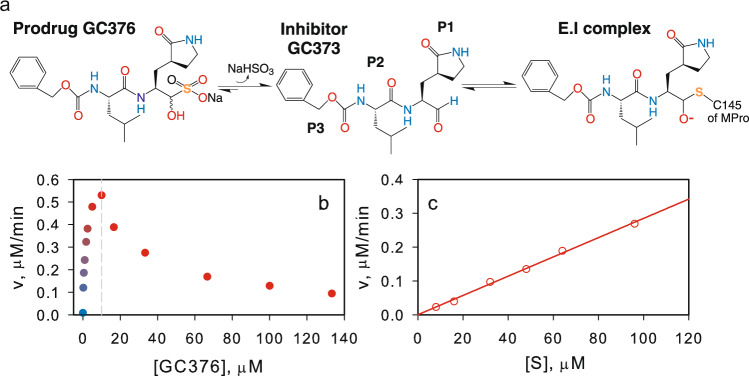
Modulation of the catalytic activity of MPro^M^ by feline coronavirus prodrug GC376. **a** Chemical structure of GC376 and steps in its binding to the active site of 3C and 3CL proteases^40,41^. **b** Catalytic activity of 10 µM MPro^M^ as a function of increasing GC376 concentration. **c** A plot of the rate *vs* substrate concentration at a final concentration of 10 µM MPro^M^ and 10 µM GC376.

Several clinical drugs have been repurposed for developing rapid therapeutic intervention of COVID-19^35,36,40,41^. Prodrug GC376 elicits a broad-spectrum activity against human and animal coronaviruses including the recent SARS-CoV-2 MPro^WT^ and its replication^40–44^. In aqueous medium, the prodrug disproportionates to a sulfite ion and the aldehyde GC373 (Fig. 5a). GC373 inhibits MPro by reversibly binding and forming a covalent bond between the sulfur of C145 and the carbonyl carbon of GC373 to yield hemithioacetal, a transition state analog. Henceforth, GC376 and GC373 are used herein interchangeably. The 3D structures of MPro^WT^-GC373 complexes were described recently^40,41^. The prodrug GC376 was chosen to examine its interaction with MPro^M^ because it is thermally more stable than other known inhibitor complexes by as much as 10.3 °C indicative of its higher affinity^40,45^.

Surprisingly, the rate of hydrolysis catalyzed by 10 µM MPro^M^ increases with the increasing inhibitor concentration reaching a maximum at 10 µM followed by a decrease above 10 µM inhibitor (Fig. 5b). At a final concentration of 10 µM each of GC376 and MPro^M^, K_m_ is much larger than the solubility limit of the substrate, and the observed first-order rate constant v_max_/K_m_ is (1.3 ± 0.2) × 10^−3 ^min^−1^ in buffer B at 28 °C is obtained from the linear plot of rate *vs* [S] (R = 0.9984), shown in Fig. 5c.

### Modulation of the M-D equilibrium of MPro^M^ and competitive inhibition by GC376

**Fig. 6:**
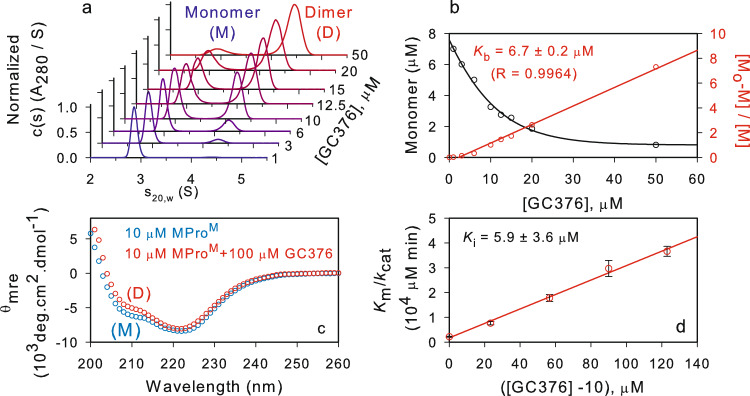
Modulation of the M-D equilibrium of MPro^M^ and competitive inhibition by GC376. **a** SV-AUC absorbance c(s) distribution of MPro^M^ (6–7 µM) in the presence of increasing GC376 concentration ranging from 1-50 µM. **b** Plot of the monomer amount *vs* inhibitor concentration (black trace) from data derived from (A). Estimation of the K_b_ for the binding of GC376 to MPro^M^ (red trace). **c** CD spectra of 10 µM MPro^M^ in the absence (blue) and presence (red) of 100 µM inhibitor GC376. **d** A plot of the K_m_/k_cat_ *vs* increasing concentration of GC376 at a final concentration of 10 µM MPro^M^. Error values indicate a standard deviation of data points recorded 4 times in duplicate (Supplementary Table 2).

Since the rate of MPro^M^-catalyzed hydrolysis displays a second-order dependency on the enzyme concentration indicating a protein dimer is required for catalytic activity, we suspected that GC376 influenced the M-D equilibrium. A series of SV analyses were carried out in the presence of varied inhibitor concentrations. Figure 6a shows SV absorbance c(s) distributions of 6–7 µM MPro^M^ at increasing concentrations of GC376. In addition to the monomeric form of the protein, a second species corresponding to the dimer form (4.47 S, 58 kDa) is observed. The amount of the dimer form increases with a corresponding decrease in the monomer form indicating a dynamic equilibrium. Figure 6b (black line) shows the decrease in the amount of the monomeric species with increasing GC376 concentration. The data can be fitted to the equation [M] + [I] ⇋ [MI], assuming a single binding site. A plot of {[M_o_]-[M]} vs [M] is linear (Fig. 6b, red trace), where [M_o_] and [M] denote the total protein and monomer concentrations, respectively. This result indicates that both the active sites of the dimer are functional and equivalent with a calculated K_b_ of 6.7 ± 0.2 µM. CD spectra (Fig. 6c) at 10 µM MPro^M^ in the absence (monomer) and 10-fold molar excess GC376, where MPro^M^ is ~90% dimeric, are nearly identical indicating a folded monomer with a secondary structure that resembles the dimer, which is consistent with earlier observations reported for SARS-CoV main protease by CD and NMR^7,21,22,31^.

**Fig. 7:**
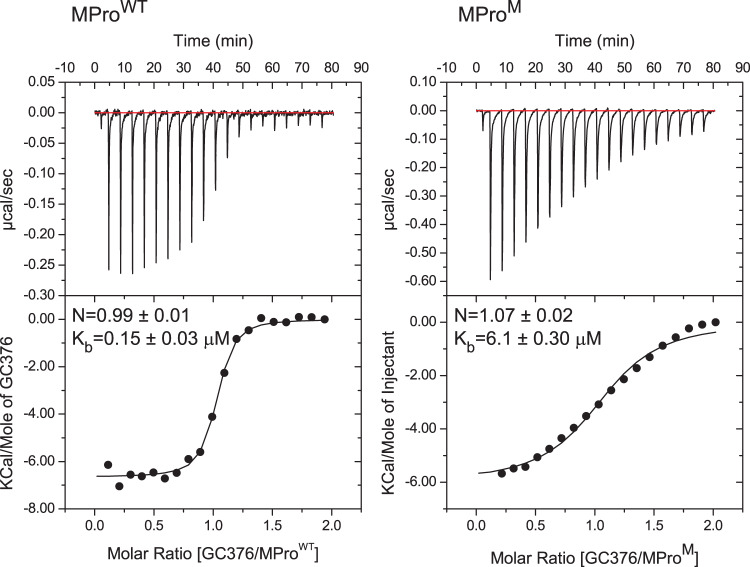
Binding isotherm of GC376 to MPro^WT^ and MPro^M^. Titrations were carried out with 30 µM MPro^WT^ and 98 µM MPro^M^ (in the cell) *vs* 300 µM and 1 mM GC376 (in the syringe), respectively, in buffer C at 28 °C. Thermodynamic parameters are listed in Supplementary Table 1.

The binding constant of GC376 to MPro^M^ was also determined by isothermal titration calorimetry (ITC). The isotherms are shown in Fig. 7 for MPro^WT^ and MPro^M^ and the binding constants and thermodynamic parameters are listed in Supplementary Table 1. The titration fits a single site binding model with nearly a 1:1 stoichiometry of MPro^M^ to inhibitor and an estimated K_b_ of 6.1 ± 0.3 µM in buffer C at 28 °C consistent with the estimated K_b_ value determined by SV-AUC analysis. The binding constant of GC376 to MPro^M^ is 41 times larger relative to MPro^WT^ which is indicative of the MPro^WT^/GC376 complex being more stable by about 2.2 kcal/mol.

**Fig. 8:**
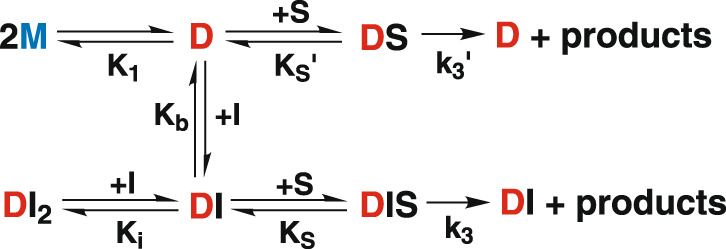
Mechanism of activation and inhibition of MPro^M^ by GC376. M, D, S, I, DS, DI, DI_2_, DIS denote monomer, dimer, substrate, inhibitor, dimer-substrate complex, dimer-inhibitor complex, dimer bound to 2 inhibitors, dimer bound to 1 inhibitor and 1 substrate, respectively.

Scheme in Fig. 8 is proposed to account for the rise and fall in the catalytic activity of MPro^M^ upon increasing the inhibitor concentration (Fig. 5b).

The second-order dependency of the rate of hydrolysis on MPro^M^ concentration points to the presence of undetectable amounts of dimer by SV-AUC up to 90 µM (Fig. 2b). The concomitant increase of the dimer population and catalytic activity upon binding GC373 further supports the requirement of a dimer for catalytic activity. Importantly, the binding of the transition state analog GC373 requires a functional active site. X-ray crystallographic and NMR solution studies of MPro^WT^-GC373 complex show that GC373 reversibly and covalently modifies the active site sulfur atom of the nucleophilic thiol of Cys145 by forming a hemithioacetal^40,41^. Therefore, the two active sites of a dimer must be equivalent and simultaneously functional contrary to the notion suggested for SARS-CoV main protease dimer that only one protomer is active at a time^26,46^. Thus, GC373 binds to a small population of the dimer leading to a shift in the equilibrium composition favoring the dimer. For MPro^M^ in the presence of GC373, the catalytically reactive species is a dimer containing one molecule of GC373 bound to one of the two active sites (DI) leaving the other available for catalytic function. It can be shown from the scheme that (see Supplementary Note 2):

4 $$v=\frac{{K}_{3}\left[S\right]\left\{\left[{M}_{o}\right]-\left[M\right]\right\}}{2{K}_{s}\left\{1+\frac{\left[I\right]}{{K}_{i}}\right\}+[S]}$$

where M_o_ is the total protein and M is the amount of the monomeric form.

The values of [M] at various concentrations (M_o_) are quantified by SV-AUC from the results shown in Fig. 6a, b. The K_i_ value is determined by evaluating inhibition at concentrations of GC376 at 10 µM and above. Because the maximal catalytic activity of MPro^M^ in the presence of GC376 is at 10 µM, the observed K_m_/k_cat_ values were plotted versus ([I] – 10) (see Fig. 6d and Supplementary Table 2). The calculated K_i_ of 5.9 ± 3 µM is within the experimental error to the values determined by SV-AUC and ITC. Because GC373 is known to bind reversibly^40,41^ and a single binding isotherm is observed in ITC both for MPro^WT^ and MPro^M^, GC373 must bind to the same form of the protein at the active site of MPro^M^ and compete with the substrate. Since k_cat_/K_m_ for MPro^WT^ is k_3_/K_s_, the value of k_3_/K_s_, i.e., the intrinsic catalytic activity for MPro^M^-catalyzed hydrolysis is calculated using Eq. 5.

5 $$\left(\frac{{v}_{{\max }}}{{K}_{m}}\right){obs}=\frac{{k}_{3}\left\{\left[{M}_{o}\right] \! - \! \left[M\right]\right\}}{2{K}_{s}\left\{1+\frac{\left[I\right]}{{K}_{i}}\right\}}$$

From the observed V_max_/K_m_ of 3.3 × 10^−3 ^min^−1^, the measured K_i_ of 6.2 µM, [M] value of 5 µM determined by SV-AUC at [M_o_] and GC376 concentration at 10 µM each, k_3_/K_s_ for MPro^M^-catalyzed hydrolysis with one molecule of GC376 bound to one of the active sites is 3.4 × 10^−3 ^µM^−1^ min^−1^ (Table 1). Since k_3_/K_s_ is the intrinsic catalytic activity for MPro^M^, it is possible to calculate the M-D equilibrium for MPro^M^ from Eq. 3 and the expression:

6 $$\frac{{k}_{{cat}}}{{K}_{m}}={k}_{3}/{K}_{1}{K}_{s}$$

K_1_ is calculated to be ~6600 µM. This allowed us to calculate the amount of the protein in the dimer form {[M_o_]-[M]} at each protein concentration. A plot of v *vs* {[M_o_]-[M]} is linear (R = 0.9992, Supplementary Fig. 3) as expected from Eq. 1. The calculated K_1_ for MPro^M^ is 5077 times larger than that of MPro^WT^ corresponding to 5.1 kcal/mol.

## Discussion

The results presented above indicate that MPro^M^ adopts a tertiary fold like MPro^WT^ (see refs. ^40,41^) as shown by its catalytic activity and ability to bind GC373 and form a transition state analog at the active site, and by the CD spectrum. The observed second-order dependency of the rate of MPro^M^-catalyzed hydrolysis, and the increase in catalytic activity upon inhibitor binding accompanied by dimer formation are *prima facie* evidence indicating that dimerization is required for mature-like catalytic activity. The kinetic parameters in Table 1 show that the catalytic activity of MPro^WT^ is higher than that of MPro^M^ in the absence and presence of GC373. For MPro^WT^- and MPro^M^-catalyzed hydrolysis, k_cat_ and k_cat_/K_m_ are k_3_ and k_3_/K_s_, respectively, and thus, they can be directly compared. MPro^WT^ k_cat_ is 56 times larger than that of MPro^M^ (see Table 1) corresponding to 2.4 kcal/mol of free energy which is the additional free energy provided by the MPro^WT^ to stabilize the transition state of the hydrolytic reaction. Similarly, MPro^WT^ k_3_/K_s_ is 120 times larger than that of MPro^M^ corresponding to 2.9 kcal/mol. Since MPro^M^ is properly folded and has catalytic activity, it appears that the catalytic efficiency is also linked to the thermodynamic stability of the dimer form as seen from the comparison of the binding constants of GC373 to MPro^WT^ and MPro^M^ which reflect the difference in the thermodynamic stability of both complexes. The binding constant of GC373 (K_b_) to MPro^M^ is 41 times larger than that for MPro^WT^ (see Table 1) indicating that MPro^WT^ complex is more stable than that of MPro^M^ by 2.2 kcal/mol. In contrast, in the absence of GC373, the E290A and R298A mutations destabilize the dimer form of the enzyme by 5.1 kcal/mol. Thus, these results indicate that enzymatic activity is highly dependent on thermodynamic stability of the dimeric form of the enzyme.

The result and conclusions presented above are consistent with published reports^31,47^. Lin et al. observed a clear correlation between k_cat_/K_m_ and the rate constant for dimer dissociation (k_off_), as well as the M-D equilibrium constant, using MPro constructs with N-terminal deletions and single or double substitution mutations which affect dimer formation of SARS-CoV^31^. Increasing both, the k_off_ and M-D dissociation constant (K_d_), lead to decreased catalytic activities. The enhanced catalytic activity with increasing inhibitor concentration accompanied by a rise in the abundance of the dimer form (see Figs. 5b and 6A) indicates that binding of the inhibitor to the dimer form of a predominantly monomeric protein changes the equilibrium composition favoring the dimer and thereby establishing the proper geometry of the active site for catalytic activity. We note that dimerization of MPro^M^ does not restore the full catalytic activity (Table 1) probably due to a larger k_off_ for MPro^M^, compared to that of MPro^WT^. Silvestrini et al. have identified two groups of compounds that alter the monomer-dimer composition of MPro^WT^ by interacting with the dimer interface^47^. The first group shows the expected strong inhibition of enzymatic activity triggered by the dissociation of the dimer, whereas the second group displays the opposite behavior. The contrary effect of the second group is explained in terms of the presence of a fraction of the protein in the dimer form for which both the inhibitor and the substrate compete. The binding of the substrate to the active sites of the dimer form produces the observed catalytic activity^47^. This observation is consistent with our results that binding the transition state analog to the enzyme favors the dimer form of the enzyme leading to enhanced catalytic activity.

It is well established that the active site of an enzyme is complementary to the transition state of a reaction and not to the substrate, and that the resulting binding energy is utilized for lowering and stabilizing the transition state of a reaction^48^. The mutations E290A and R298A in MPro^M^ destabilize the dimeric form of the enzyme. Consequently, they distort the complementarity between the transition state of the hydrolytic reaction and the active site leading to a loss of 5.1 kcal/mol of stabilization energy to the transition state of the hydrolytic reaction. In accordance, crystalline structures of the monomeric main protease constructs G11A, S139A and R298A of SARS-CoV show similar major reorganization of the active site including the P1 binding site of the substrate and the catalytic loop comprising the oxyanion hole constituting the weakly-functional catalytic machinery^20,27–29,32^. Thus, the collapsed loop conformation is likely to be an attribute of the monomeric MPro and independent of the position or type of mutation leading to dimer dissociation. It is worth noting that the crystal structure is a single static conformation, i.e., one of many conformations in solution, one of which, albeit at a much lower abundance, forms a dimer to provide the observed catalytic activity of MPro^M^. As shown above, the rate of hydrolysis of the peptide substrate displays a second-order dependency on MPro^M^ concentration indicating the presence of a finite amount of dimeric form of MPro^M^ which is insufficient to be detected by optical methods. The largest difference between MPro^M^ and MPro^WT^ is in their M-D equilibrium constant which reflects the difference in overall catalytic activity. Thus, structure-based design of non- or un-competitive inhibitors that bind to a different form or site of the enzyme and interfere with dimer formation could prove to be an effective alternative strategy to impair catalytic activity^26^. In particular, the dimer interface may be targeted to identify a compound that directly interferes with dimer formation. Since MPro^M^ is predominantly monomeric and displays measurable catalytic activity as well as activated and inhibited by a competitive inhibitor of MPro^WT^ by modulating the monomer-dimer equilibrium, it may be a valuable tool in identifying such inhibitors.

## Methods

### Expression and purification

The coding sequence of the main protease (MPro) of SARS-CoV-2 (GenBank ID: MN908947.3) bearing the substitution mutations E290A and R298A (MPro^M^, where M denotes monomer, Supplementary Fig. 1a) and the flanking 25 residues of nsp4 at the N-terminus and a 6His-Tag at the C-terminus was synthesized and cloned into pJ414 vector (ATUM, Newark, CA). The plasmid was transformed into BL21-DE3 cells (Agilent) and induced for expression at 0.7-0.8 optical density with 1 mM isopropyl β-D-1-thiogalactopyranoside for 3 h. The processed MPro^M^ was purified from the cell lysate by nickel-affinity chromatography (NAC). The bound fraction was subjected to isocratic fractionation on Superose-12 column (step 1, Cytiva Life Sciences) and HRV-3C protease cleavage (step 2, purchased from Sigma-Aldrich) followed by repeating NAC and step 1 in a final buffer of 25 mM Tris-HCl, pH 7, 150 mM NaCl and 1 mM TCEP (buffer A). The full-length wild type (MPro^WT^) was expressed and purified similar in strategy to that described previously^3^ except for substituting the fusion partner GST with maltose binding protein (MBP) followed by a 36 amino acid spacer sequence corresponding to the immunoglobulin binding domain B1 of protein G^49^. Peak fractions were concentrated (5–6 mg/ml) and stored in aliquots at −20 °C and for long term storage at −80 °C. Purity was verified both by SDS-PAGE and electrospray ionization mass spectrometry.

### Enzyme kinetics

Activity assays using the FRET substrate Dabsyl-KTSAVLQ/SGFRKM-E(Edans)-NH2^3,35–37^, where (/) denotes the scissile peptide bond, were carried out in a total volume of 100 µl in 25 mM Tris-HCl, pH 7, 50 mM NaCl and 1 mM TCEP (buffer B) at 28 °C. Assays were initiated by adding the reaction mixture (95 µl) with or without the inhibitor GC376 to 5 µl of substrate in 100% DMSO kept in the microplate well (Reference 655809, Greiner bio-one). When involving inhibitor, the reaction mixture was incubated for a period of 10 min prior to initiating the reaction with substrate. The excitation and emission wavelengths were set to 336 nm and 490 nm, respectively, and the increase in emission fluorescence intensity was recorded 2–4 times per data point depending on the duration of the data collection as a function of time in a Tecan Infinite M plex microplate reader. After background correction of the average of no enzyme negative controls, concentration of substrate cleaved was determined from a EDANS standard plot and instrument specific inner filter correction values were applied as described^3,50^ prior to calculating the kinetic parameters as described below. The substrate was custom synthesized (Biomatik, Ontario, Canada) and GC376 was purchased from Selleckchem, Houston, TX. ΔG was calculated according to the equation ΔG = -RTlnK. The free monomer concentrations in the absence of the inhibitor are calculated from the equations: K_1_ = [M]^2^/[D] and [M_o_] = [M] + 2[D].

### Statistics and reproducibility

The FRET substrate is highly sensitive and widely used to assess MPro activity^3,35–37^. Stock solutions of the enzyme, FRET substrate (5 mM in 100% DMSO) and GC376 in (100 mM in 10% DMSO) stored in aliquots at −20 °C were freshly diluted prior to the experiment. The enzyme and inhibitor solutions were kept on ice and the substrate at room temperature while setting up the experiment. The solubility limit of the enzyme, substrate and the inhibitor were verified under the assay buffer conditions. The solubility of the substrate and its products were also monitored during the assay. The reproducibility of enzyme kinetics was tested at least 2-3 times with freshly prepared enzyme and stock solutions of the substrate and inhibitor. Once this was determined to provide consistent reaction rates within an error limit of 5%, the final experiment for the data displayed in the manuscript was carried out in duplicate and 2–4 reads per well for each time point. The mean of the data points was used for fitting. Measurements were processed using SigmaPlot (Systat) by fitting Michaelis-Menten (non-linear) or linear equations to data to calculate the kinetic parameters, standard deviations by the least squares method and multiple correlation coefficients (R). Also, the binding constant of the inhibitor to the enzyme was determined by SV-AUC and ITC using the same stock solutions of enzyme and inhibitor. The binding constants of the inhibitor to the enzyme obtained by the three independent methods were within the reported experimental errors.

### Sedimentation velocity analytical ultracentrifugation (SV-AUC)

Protein stock solutions maintained in buffer A were diluted to a final concentration ranging from 10-90 µM. Samples containing the inhibitor GC376 were prepared using a 1 mM stock solution of GC376 in buffer B to achieve the desired protein and GC376 ratios in buffer B and a final concentration of 0.1% DMSO.

Sedimentation velocity experiments were conducted at 50,000 rpm and 25 °C on a Beckman Coulter ProteomeLab XL-I analytical ultracentrifuge following standard protocols^51^. Samples were loaded in 2-channel centerpiece cells and scans were collected using both the absorbance (280 nm) and Rayleigh interference (655 nm) optical detection systems. Sedimentation data were time-corrected and analyzed in SEDFIT 16.1C^52^ in terms of a continuous c(*s*) distribution of Lamm equation solutions. Solution densities ρ, solution viscosities η, and protein partial specific volumes were calculated in SEDNTERP^53^.

### Circular Dichroism

CD spectra were recorded in buffer B at 25 °C on a JASCO J-810 spectropolarimeter using Spectra Manager software version 2 (Jasco Analytical Instruments, Easton, MD) and a 0.1 cm pathlength cell. Spectra were processed using the same software.

### Size exclusion chromatography with multi-angle light scattering (SEC-MALS)

Molecular mass of MPro^M^ and MPro^WT^ was estimated by analytical SEC with in-line MALS (DAWN Heleos-II, Wyatt Technology Inc., Santa Barbara, CA), refractive index (Optilab T-rEX, Wyatt Technology Inc.) and UV (Waters 2487, Waters Corporation, Milford, MA) detectors. Sample was applied onto a pre-equilibrated Superose-12 column (1.0 × 30 cm) and eluted at a flow rate of 0.5 mL/min in buffer A at 25 °C. Molecular mass was calculated using the Astra software provided with the instrument.

### Isothermal Titration Calorimetry (ITC)

Purified proteins were diluted from a stock solution and dialyzed extensively against buffer C (25 mM Tris-HCl, pH 7.2, 20 mM NaCl and 1 mM TCEP). Concentrations were estimated after dialysis based on their 280 nm absorbance. A stock solution of GC376 in 10% DMSO was diluted in buffer C to the desired concentration. Titrations were performed at 28 °C on iTC200 microcalorimeter (Malvern Instruments Inc., Westborough, MA). A control titration of buffer with inhibitor showed negligible response. Data were processed using the Origin software provided with the instrument. For competitive inhibitors that bind at only one site, the binding constant {K_binding_ (K_b_) = 1/K_a_} is equivalent to the inhibition constant measured by enzyme kinetics (K_i_).

### Reporting summary

Further information on research design is available in the Nature Research Reporting Summary linked to this article.

## Supplementary information

Supplementary Information

Description of additional supplementary files

Supplementary data 1

Supplementary data 2

Reporting Summary

## Data Availability

Source data files are provided in Supplementary Data 1 and 2.
