# Supplementary material for: Modulation of the monomer-dimer equilibrium and catalytic activity of SARS-CoV-2 main protease by a transition-state analog inhibitor
Source: Commun Biol. 2022 Mar 1;5:160. doi: 10.1038/s42003-022-03084-7 (PMC8888643; doi:10.1038/s42003-022-03084-7)
Supplement: Supplementary file 1 — Supplementary Information [file 42003_2022_3084_MOESM1_ESM.docx]

Supplementary information

Modulation of the monomer-dimer equilibrium and catalytic activity of SARS-CoV-2 main protease by a transition-state analogue inhibitor

Nashaat T. Nashed,^1^ Annie Aniana,^1^ Rodolfo Ghirlando,^2^ Sai Chaitanya Chiliveri,^1^ and John M. Louis,^1,^*

^1^Laboratory of Chemical Physics, National Institute of Diabetes and Digestive and Kidney Diseases, National Institutes of Health, Bethesda, MD 20892, USA

^2^Laboratory of Molecular Biology, National Institute of Diabetes and Digestive and Kidney Diseases, National Institutes of Health, Bethesda, MD 20892, USA

* Correspondence to John M. Louis (johnl@niddk.nih.gov)

**Supplementary Table 1.** **Binding affinity of GC376 to MPro^M^ relative to MPro^WT^ as determined by ITC**

| Construct | N | K_b_ (µM) | ΔH kcal/mol | ΔS cal/mol/K | -TΔS kcal/mol | ΔG kcal/mol |
| --- | --- | --- | --- | --- | --- | --- |
| MPro^WT^ | 0.99 ± 0.01 | 0.15 ± 0.03 | -6.7 ± 0.1 | 9.1 | -2.7 | -9.4 |
| MPro^M^ | 1.07 ± 0.02 | 6.13 ± 0.30 | -6.0 ± 0.2 | 3.9 | -1.2 | -7.2 |

**Supplementary Table 2. Competitive inhibition of MPro^M^ by GC376**

| ([GC376] -10), µM | *K*_m_/*k*_cat_ (10^4^ µM min) | SD |
| --- | --- | --- |
| 0 | 0.217 | 0.019 |
| 23.3 | 0.764 | 0.066 |
| 56.6 | 1.780 | 0.136 |
| 90 | 2.970 | 0.326 |
| 123 | 3.660 | 0.203 |

Supplementary Fig. 1.

MPro^M^

10 20 30 40 50 60

SGFRKMAFPS GKVEGCMVQV TCGTTTLNGL WLDDVVYCPR HVICTSEDML NPNYEDLLIR

70 80 90 100 110 120

KSNHNFLVQA GNVQLRVIGH SMQNCVLKLK VDTANPKTPK YKFVRIQPGQ TFSVLACYNG

130 140 150 160 170 180

SPSGVYQCAM RPNFTIKGSF LNGSCGSVGF NIDYDCVSFC YMHHMELPTG VHAGTDLEGN

190 200 210 220 230 240

FYGPFVDRQT AQAAGTDTTI TVNVLAWLYA AVINGDRWFL NRFTTTLNDF NLVAMKYNYE

250 260 270 280 290 300

PLTQDHVDIL GPLSAQTGIA VLDMCASLKE LLQNGMNGRT ILGSALLED**A** FTPFDVV**A**QC

306

SGVTFQ


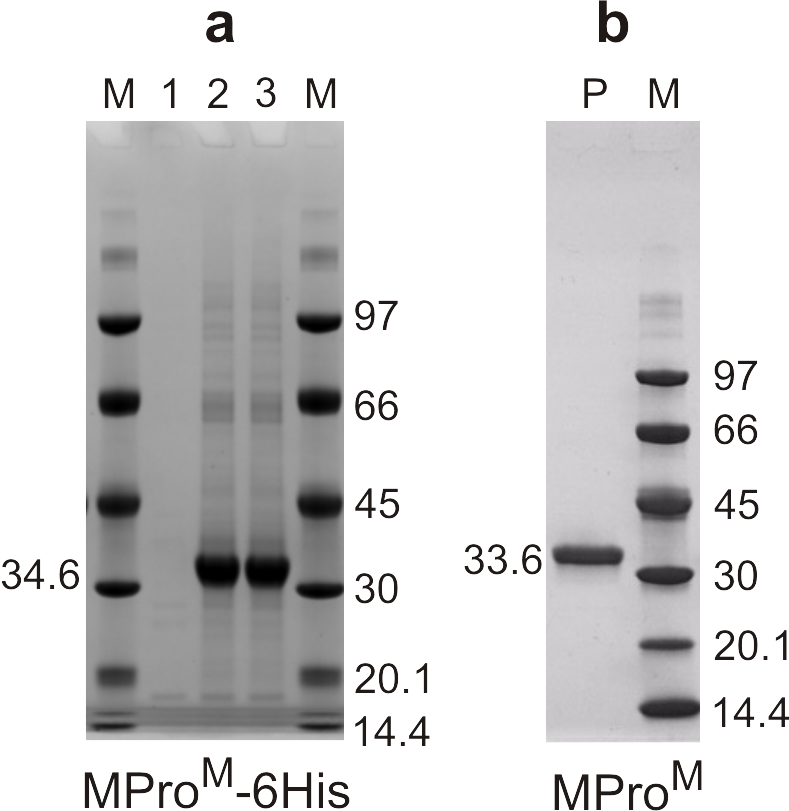


**Supplementary Fig. 1.** **Expression and purification of MPro^M^.** (Top) Amino acid sequence of MPro^M^ bearing the mutations E290A and R298A (shown in red). The C-terminal 6His-Tag was removed using HRV-3C protease as described^1^. (a) Accumulation of processed MPro^M^-6His upon expression of its precursor ^+25^MPro^M^-6His. Cell extracts derived from 20 mL culture were subjected to Nickel-affinity purification followed by SDS-PAGE. Lane 1 denotes the uninduced, and lanes 2 and 3, induced for 90 and 180 min, respectively. M denotes molecular weight standards in kDa. MPro^M^-6His migrates at an expected size of 34.6 kDa. (b) Purified (P, 33.6 kDa) MPro^M^ following 6His-Tag removal.

Supplementary Fig. 2.


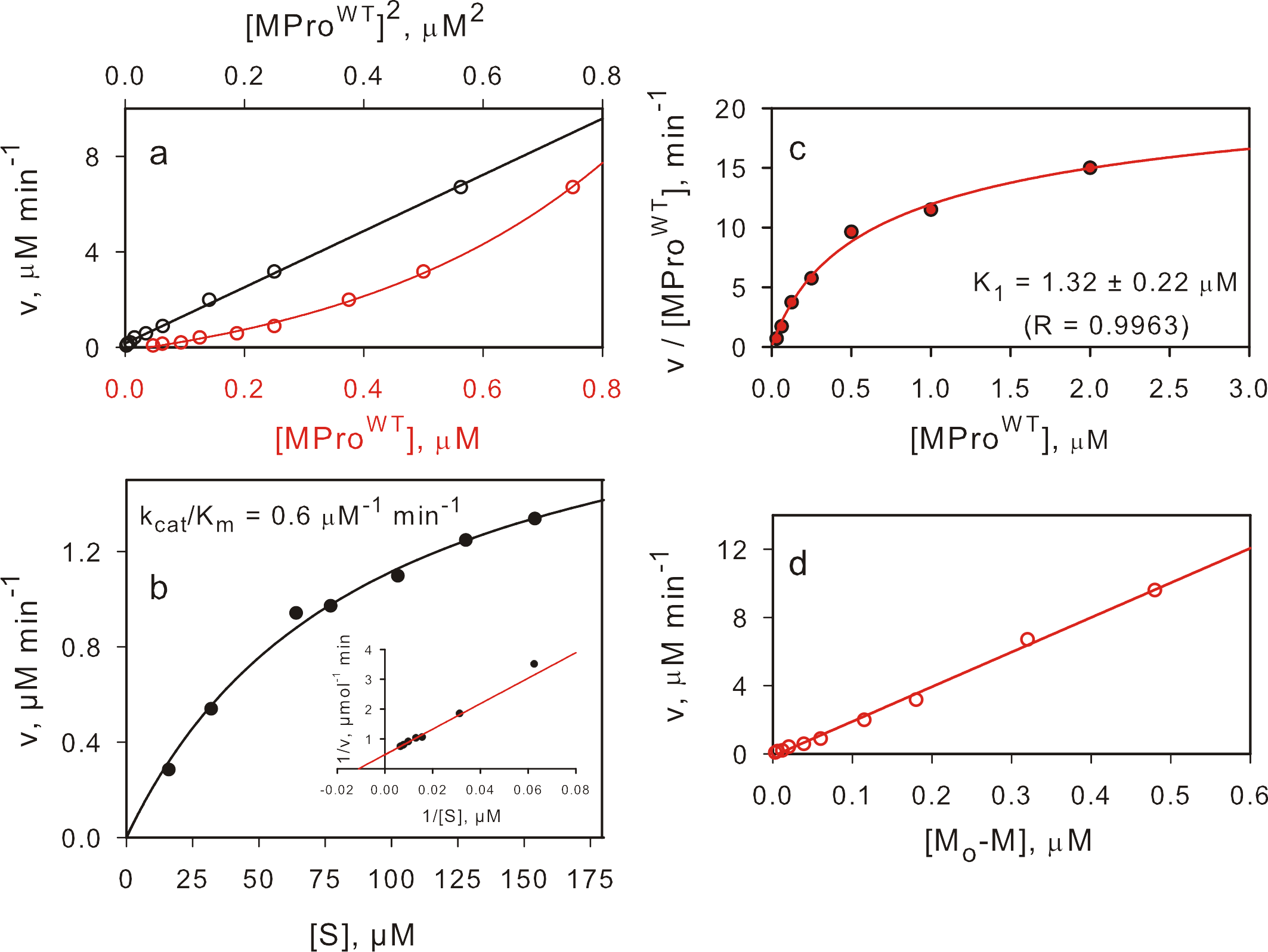


**Supplementary Fig. 2.** **Kinetics of MPro^WT^.** (a) Non-linear relationship between the rate of substrate hydrolysis *vs* the protein concentration (red line), and the linear relationship between the rate of hydrolysis of substrate *vs* the square of the protein concentration (black line). (b) Michaelis-Menten and Lineweaver-Burk (inset) plots for hydrolysis of substrate by 200 nM MPro^WT^. (c) Kinetic determination of the dimer dissociation constant from the dependence of rate for hydrolysis of substrate on enzyme concentration^2, 3^. Equation 1 from reference 2 was fitted to the mean of a duplicate data set by least squares method. (d) The relationship between the rate of hydrolysis of substrate at 200 µM and the protein concentration (as dimer). Assays were performed in buffer B at 28 ̊C. Data were analyzed and plotted using SigmaPlot (Systat).

Supplementary Fig. 3.


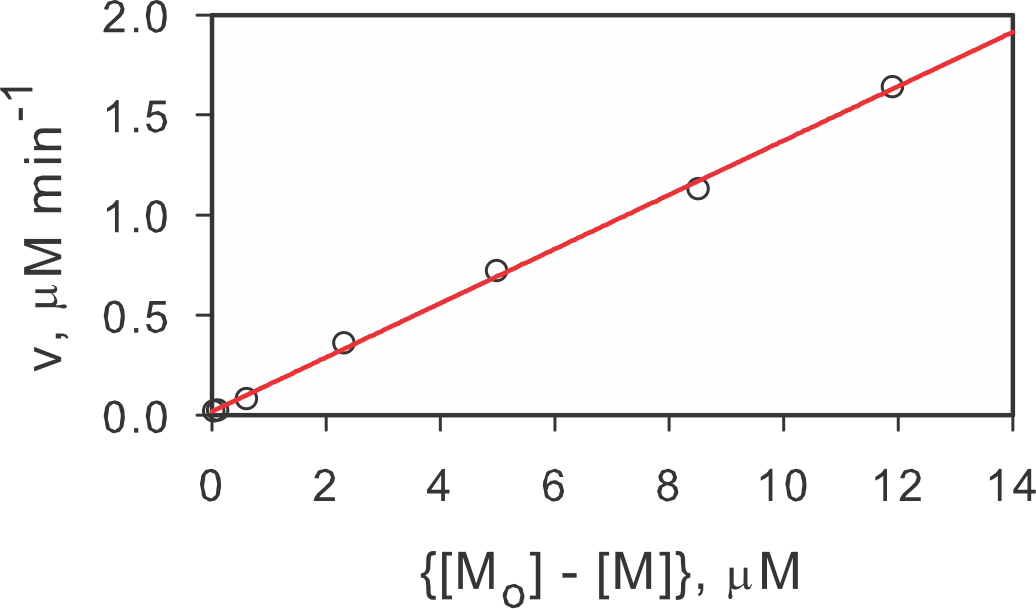


**Supplementary Fig. 3.** **Rate of MPro^M^-catalyzed hydrolysis vs protein concentration in the dimer form.** See equation 1 in main text.

Supplementary Note 1:


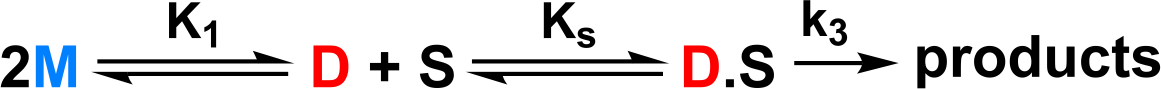


K_1_ = [M]^2^/[D] (1)

K_S_ = [D][S]/[DS] (2)

[DS] = [M]^2^[S]/K_1_K_S_ (3)

Since each dimer has two equivalent and independently functional active sites,

v = 2k_3_[DS] (4)

v = 2k_3_[M]^2^[S]/K_1_K_S_ (5)

[M_o_] = [M] + 2[D] + 2[DS] (6)

= [M] + 2[M]^2^/K_1_ + 2[M]^2^[S]/K_1_K_S_ (7)

Where M_o_ is the total protein concentration. Rearranging equation 7

[M]^2^ = K_1_K_S_{[M_o_] – [M]}/2{K_S_ + [S]} (8)

v = k_3_ {[M_o_] –[M]}[S]/{K_s_ + [S]} (9)

Supplementary Note 2:


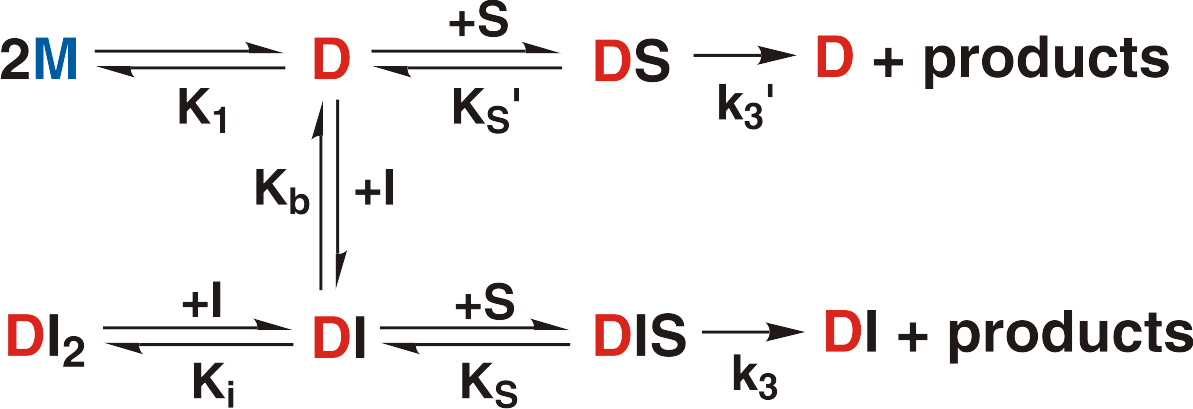


v = k_3_’ [DS] + k_3_’ [DIS] (1)

Since no D is observed in the absence of inhibitor up to 90 µM, [DI] >>> [D], k_3_ [DIS] >>> k_3_’ [DS] and only one active site per dimer is available for catalytic activity,

v = k_3_ [DIS] (2)

[M_o_] = [M] + 2[DI] + 2[DIS] + 2[DI_2_] (3)

K_1_ = [M]^2^/[D] (4)

K_b_ = [D][I]/[DI] = [M]^2^[I]/K_1_[DI] (5)

K_i_ = [DI][I]/[DI_2_] = [M]^2^[I]^2^/K_1_K_b_[DI_2_] (6)

K_s_ = [DI][S]/[DIS] = [M]^2^[I][S]/K_1_K_b_[DIS] (7)

Substituting value [DIS] in equation 2,

v = k_3_[M]^2^[I][S]/K_1_K_b_K_S_ (8)

Substituting the value of [DI], [DI_2_], and [DIS] in equation 3 from equations 4-7,

[M_o_] = [M] + 2[M]^2^[I]/K_1_K_b_ + 2[M]^2^[I]^2^/K_1_K_b_K_i_ + 2[M]^2^[I][S]/K_1_K_b_K_S_ (9)

{[M_o_] - [M]} = 2[M]^2^[I]{1/K_1_K_b_ + [I]/K_1_K_b_K_i_ + [S]/K_1_K_b_K_S_} (10)

[M]^2^ = {[M_o_] - [M]}/2{[I]1/K_1_K_b_ + [I]/K_1_K_b_K_i_ + [S]/K_1_K_b_K_S_} (11)

Substituting [M]^2^ in equation 8 by equation 11,

v = k_3_[S]{([M_o_] - [M])/2}/K_1_K_b_K_S_{1/K_1_K_b_ + [I]/K_1_K_b_K_i_ + [S]/K_1_K_b_K_S_} (12)

v = k_3_[S]{([M_o_] - [M])/2}/{K_S_(1 + [I]/K_i_) + [S]} (13)

k_cat_/K_m_ = k_3_/K_S_{1 + [I]/K_i_} (14)

**References**

1. Xue X*, et al.* Production of authentic SARS-CoV M(pro) with enhanced activity: application as a novel tag-cleavage endopeptidase for protein overproduction. *J Mol Biol* **366**, 965-975 (2007).

2. Sayer JM, Agniswamy J, Weber IT, Louis JM. Autocatalytic maturation, physical/chemical properties, and crystal structure of group N HIV-1 protease: relevance to drug resistance. *Protein Sci* **19**, 2055-2072 (2010).

3. Todd MJ, Semo N, Freire E. The structural stability of the HIV-1 protease. *J Mol Biol* **283**, 475-488 (1998).
