# Supplementary material for: Modulation of the monomer-dimer equilibrium and catalytic activity of SARS-CoV-2 main protease by a transition-state analog inhibitor
Source: Commun Biol. 2022 Mar 1;5:160. doi: 10.1038/s42003-022-03084-7 (PMC8888643; doi:10.1038/s42003-022-03084-7)
Supplement: Supplementary file 2 — Description of additional supplementary files [file 42003_2022_3084_MOESM2_ESM.pdf]

Description of additional supplementary files

File name: Supplementary data 1
Description: Source data for all main figures

File name: Supplementary data 2
Description: Source data for all supplementary information figures
